# Range-wide neutral and adaptive genetic structure of an endemic herb from Amazonian Savannas

**DOI:** 10.1093/aobpla/plaa003

**Published:** 2020-01-31

**Authors:** Amanda R Silva, Luciana C Resende-Moreira, Carolina S Carvalho, Eder C M Lanes, Mabel P Ortiz-Vera, Pedro L Viana, Rodolfo Jaffé

**Affiliations:** 1 Universidade Federal Rural da Amazônia/Museu Paraense Emílio Goeldi, Programa de Pós-graduação em Ciências Biológicas - Botânica Tropical, Belém-PA, Brazil; 2 Museu Paraense Emílio Goeldi, Programa de Capacitação Institucional (PCI), Belém-PA, Brazil; 3 Instituto Tecnológico Vale, Desenvolvimento Sustentável, Belém-PA, Brazil; 4 Universidade Federal do Pará, Instituto de Ciências Biológicas, Programa de Pós-Graduação em Genética e Biologia Molecular, Belém-PA, Brazil; 5 Universidade de São Paulo, Departamento de Ecologia, São Paulo-SP, Brazil

**Keywords:** *Brasilianthus carajensis*, conservation genomics, environmental association tests (EAT), evolutionary significant unit, genotype–environment association (GEA), single nucleotide polymorphism (SNP)

## Abstract

Conserving genetic diversity in rare and narrowly distributed endemic species is essential to maintain their evolutionary potential and minimize extinction risk under future environmental change. In this study we assess neutral and adaptive genetic structure and genetic diversity in *Brasilianthus carajensis* (Melastomataceae), an endemic herb from Amazonian Savannas. Using RAD sequencing we identified a total of 9365 SNPs in 150 individuals collected across the species’ entire distribution range. Relying on assumption-free genetic clustering methods and environmental association tests we then compared neutral with adaptive genetic structure. We found three neutral and six adaptive genetic clusters, which could be considered management units (MU) and adaptive units (AU), respectively. Pairwise genetic differentiation (*F*_ST_) ranged between 0.024 and 0.048, and even though effective population sizes were below 100, no significant inbreeding was found in any inferred cluster. Nearly 10 % of all analysed sequences contained loci associated with temperature and precipitation, from which only 25 sequences contained annotated proteins, with some of them being very relevant for physiological processes in plants. Our findings provide a detailed insight into genetic diversity, neutral and adaptive genetic structure in a rare endemic herb, which can help guide conservation and management actions to avoid the loss of unique genetic variation.

## Teaser

Rare and narrowly distributed plants are particularly sensitive to the loss and fragmentation of their natural habitats, which ultimately result in reduced genetic diversity and a weakened ability to adapt to changing environments. In this study we evaluated the genetic health of a narrowly distributed herb from Amazonian Savannas. Relying on Next-Generation Sequencing technologies we were able to take a detailed glance at a representative portion of the plant’s whole genome, assessing genetic diversity, genetic composition and adaptations to local environmental conditions. Our results provide clear guidance on how to avoid the loss of unique genetic variation in this unique plant.

## Introduction

The assessment of population genetic structure has frequently been employed to delineate conservation units (Moritz 1994; Frankham *et al.* 2017; Coates *et al.* 2018). Evolutionary significant units (ESU), broadly defined as independent demographic entities or groups exhibiting high genetic distinctiveness due to historical isolation, have traditionally been the most commonly discussed types of conservation units (Frankham *et al.* 2017; Coates *et al.* 2018). These are delimited based on neutral genetic variation (which is not subject to natural selection), and thus mainly reflect the interplay between gene flow and genetic drift (Holderegger *et al.* 2006; Funk *et al.* 2012). The advance of Next-Generation Sequencing technologies has made possible large-scale assessments of adaptive genetic variation, which can also inform management actions (Funk *et al.* 2012). For instance, genetic variation that is exposed to natural selection (and thus underlies fitness-related traits) can exhibit different spatial patterns than those shown by neutral genetic variation (Van Wyngaarden *et al.* 2017; Barbosa *et al.* 2018), and provides valuable insights on how likely individuals are to survive under certain environmental conditions and the strength of local adaptations across real landscapes (Moritz 1999; McKay *et al.* 2005; Edmands 2007; Frankham *et al.* 2017). However, while a few studies have assessed both neutral and adaptive genetic differentiation in economically important species (Moore *et al.* 2014; Candy *et al.* 2015; Batista *et al.* 2016; Van Wyngaarden *et al.* 2017; Gugger *et al.* 2018; Jaffé *et al.* 2019), less have done so in species of conservation concern (Rodríguez-Quilón *et al.* 2016; Barbosa *et al.* 2018; Martins *et al.* 2018; Borrell *et al.* 2019; Li *et al.* 2019).

Recent calls for a paradigm shift in the genetic management of fragmented populations highlight the importance of assessing both neutral and adaptive genetic variation to delineate management units (MU) and adaptive units (AU, see definitions in Funk *et al.* 2012 and Frankham *et al.* 2017), and examine the risks and benefits of separate or joint management alternatives (Frankham *et al.* 2017; Funk *et al.* 2018; Ralls *et al.* 2018). Following the recommendations made by Frankham *et al.* (2017, page 216), populations should be managed separately when they show adaptive differentiation, as the risk of outbreeding depression resulting from crossing individuals from different AU is usually high. In the absence of adaptive differentiation the risk of outbreeding depression is low, so there is no need for separate management. Genetic rescue, or the re-establishment of gene flow between populations aiming to reverse the loss of genetic diversity, is only recommended under neutral genetic differentiation without adaptive differentiation, in situations where MU are suffering from genetic erosion or inbreeding depression.

Narrowly distributed endemic species constitute prime targets for conservation genomic assessments, because they often have small population sizes and are thus more exposed to genetic drift, have lower genetic diversity, higher inbreeding and slower adaptive responses (Hamrick *et al.* 1991; Gitzendanner and Soltis 2000; Cole 2003; Leimu *et al.* 2006; Gibson *et al.* 2008; Allendorf *et al.* 2013). One example of a narrow endemism of conservation concern, *Brasilianthus carajensis* (Melastomataceae), stands out for being a monotypic plant genus from the Amazonian Savannas of the Carajás mineral province (Mota *et al.* 2015; Viana *et al.* 2016). This mountainous complex from the Eastern Amazon is composed of banded ironstone formations (known as Cangas), and displays a very distinct vegetation that resembles Montane Savannas (Mota *et al.* 2015; Silveira *et al.* 2016; de Carvalho and Mustin 2017). The acidic, shallow, nutrient-poor, metal-rich soils with high surface temperatures, characteristic of these environments (Skirycz *et al.* 2014), associated with severe drought periods (Mota *et al.* 2015), impose severe challenges for plant growth. These extreme conditions are believed to be an important force driving local endemism (Mota *et al.* 2015; Salas *et al.* 2015; Cavalcanti *et al.* 2016), which represents about 4 % of all Canga flora (Giulietti *et al.* 2019). The high concentration of iron ore has also attracted considerable attention from mining companies (Skirycz *et al.* 2014), and approximately 20 % of the areas occupied by Canga vegetation have already been lost due to conversion to mining areas or pasturelands (Souza-Filho *et al.* 2019). In addition, species from mountainous habitats are particularly threatened by climate change since future conditions are likely to reduce occurrence range and leave little or none suitable habitat (Dullinger *et al.* 2012).

**Figure 1.**
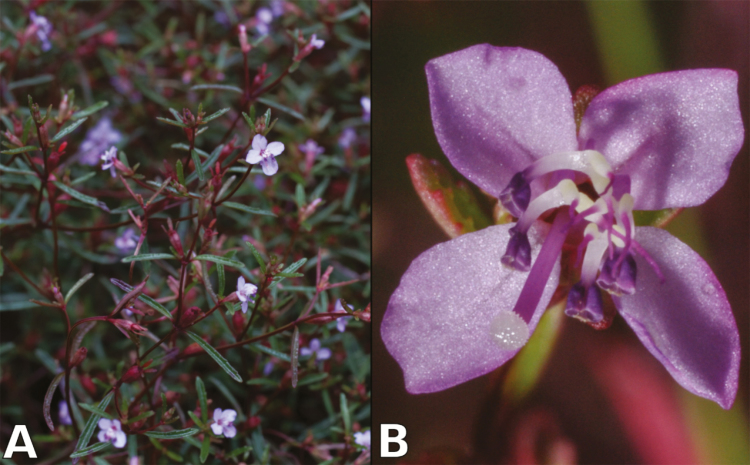
*Brasilianthus carajensis* bush (A) and flower front view (B).

*Brasilianthus carajensis* was only recently described as a new species, and is a small annual herb with four-merous flowers, wide truncate apical pore in the anthers with staminal appendages, and capsular fruit (Almeda *et al.* 2016) (Fig. 1). Fruit morphology indicates that seed dispersal is mediated by the wind, like other Melastomataceae species with capsular fruit (Renner 1989), whereas flowers are apparently pollinated by insects (Rocha *et al.* 2017). A recent landscape genomic study revealed that gene flow in this species is mainly influenced by geographic distance, terrain roughness and climate, and suggested it is resilient to habitat loss driven by mining (Carvalho *et al.* 2019). However, adaptive genetic structure has not been examined yet, and proxies for genetic diversity like nucleotide diversity and effective population size are still lacking for this species. This information is nevertheless essential to delineate MU and AU, assess the risk of inbreeding and outbreeding depression and define management actions in this rapidly changing environment (Jamieson and Allendorf 2012; Frankham *et al.* 2017; Ralls *et al.* 2018).

In this study, we used the genomic resources generated for *B. carajensis* in a previous study (Carvalho *et al.* 2019) to assess genetic diversity, neutral and adaptive genetic structure across the species’ entire distribution range. Considering the narrow and naturally fragmented distribution of *B. carajensis* across Montane Savanna highlands and the scarce life history information available, we expected to find low genetic diversity and high neutral genetic structure, mostly associated with distinct Canga plateaus. Additionally, due to the heterogeneous (Mitre *et al.* 2018) and hostile environment where the species occurs, we expected to find loci associated to environmental conditions and differing neutral and adaptive genetic structure. We identify MU and AU and discuss possible management options to prevent the loss of unique genetic variation and increase the species resilience to future environmental change.

## Materials and Methods

### Genomic data

**Figure 2.**
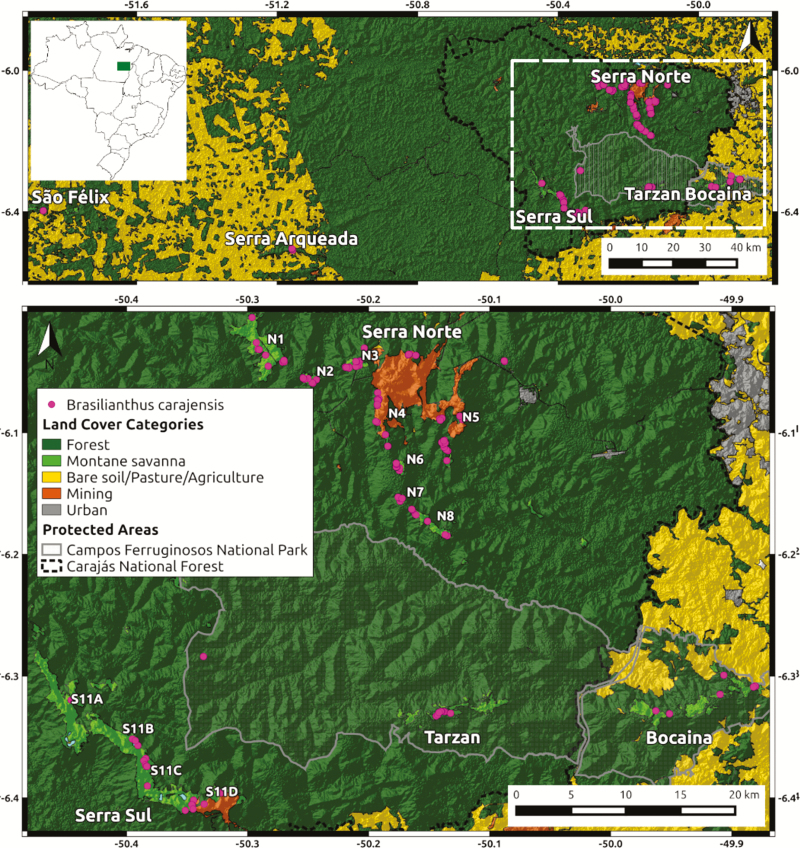
Maps of the study area showing the location of the collected samples from *Brasilianthus carajensis*. A hillshade elevation map (from USGS Earth Explorer) is shown overlaid with a land cover colour map (from Souza-Filho *et al*. 2016). Coordinates are shown in decimal degrees. While the upper map shows the full extent of our study region (and its location within Brazil), the lower map expands the area within the white-dashed square to detail the main Montane Savanna highlands and mining areas.

We used the genomic data generated by Carvalho *et al.* (2019) and publicly available in FigShare: https://doi.org/10.6084/m9.figshare.8224175.v3. Briefly, 150 individuals of *B. carajensis* were collected from all the major Montane Savanna highlands of the Carajás mineral province (SISBIO collection permit N. 48272-4), encompassing the entire species’ distribution range (Rocha *et al.* 2017) (Fig. 2). We sampled individuals separated by at least 20 m from each other to avoid collecting siblings. Vegetative branches of young plants were silica-dried at room temperature. Genomic DNA was extracted using a modified CTAB 2 % method (Doyle 1987) without phenol and subsequently purified by precipitation of polysaccharides (Michaels *et al.* 1994). DNA integrity was assessed through 1.5 % agarose gel electrophoresis, DNA concentration was quantified using the Qubit High Sensitivity Assay kit (Invitrogen) and all samples were adjusted to a final concentration of 5 ng µL^−1^. DNA samples were shipped to SNPSaurus (http://snpsaurus.com/) for sequencing and bioinformatic processing. The nextRAD libraries were prepared (Russello *et al.* 2015) and sequenced on four lanes of a HiSeq 4000 generating single-end 150 bp reads (University of Oregon). Raw data processing used custom scripts (SNPsaurus, LLC). First, reads were trimmed using *bbduk* from BBMap tools (Bushnell 2019). Second, *de novo* reference contigs were created by collecting a total of 10 million reads, evenly distributed across samples, after excluding reads with depth lower than five or higher than 700. Third, the remaining reads were aligned to each other to identify alleles and collapse haplotypes to a single representative. Finally, all reads were mapped to the reference with an alignment identity threshold of 90 % using *bbmap* (BBMap tools). Genotype calling was done using Samtools and bcftools. Alleles with a population frequency of less than 3 % were removed, and loci that were heterozygous in all samples or had more than two alleles in a sample (suggesting collapsed paralogs) were discarded.

### Genetic diversity and neutral genetic structure

Genotype data were submitted to a final quality control using VCFtools (Danecek *et al.* 2011), executed through the *r2vcftools* R package (https://github.com/nspope/r2vcftools). Loci were filtered for quality (Phred score > 50), read depth (30 – 240), linkage disequilibrium (LD, *R*^2^ < 0.6) and deviations from the Hardy–Weinberg equilibrium (HWE). For HWE filtering, the data were initially subdivided into large geographic groups (to better meet the assumptions of idealized populations), and loci showing strong deviations (*P* < 0.0001) in any of these groups were excluded. Additionally, we removed loci potentially under selection conducting a genomic scan analysis implemented in the R package LEA (Frichot and François 2015). This analysis compares single-locus estimates of population differentiation with the overall genome-wide background, while accounting for population structure. We controlled for false discovery rates by adjusting *P-*values with the genomic inflation factor (λ) and setting false discovery rates to *q* = 0.05, using the Benjamini–Hochberg algorithm (François *et al.* 2016).

The assumptions underlying commonly employed genetic clustering software like STRUCTURE (a Bayesian approach) or ADMIXTURE (based on maximum likelihood estimation) include absence of genetic drift, existence of HWE and lack of LD in ancestral populations (Frichot *et al.* 2014). These genetic clustering approaches are thus not suited to assess adaptive genetic structure, given that adaptive loci are often in LD (as they are linked to neighbouring genes under selection), and frequently depart from the HWE (Hartl and Clark 2006). We therefore employed two alternative assumption-free genetic clustering methods to assess and compare neutral and adaptive genetic structure: discriminant analysis of principal components (DAPC) (Jombart *et al.* 2010) and spatial principal component analysis (sPCA) (Jombart *et al.* 2008). Both assumption-free methods were performed using the R package *adegenet* (Jombart 2008). In DAPC, the user informs the number of clusters (in this case, ranging from 1 to 20) and genomic variation is maximized among groups, with the optimal number of clusters being inferred from the Bayesian Information Criterion (BIC). Individuals were assigned to genetic clusters based on the ancestry coefficients retrieved from DAPC analyses. In sPCA, genetic structure is estimated from scores summarizing genetic variability which also account for the geographic location of samples, thus representing the spatial pattern of genetic variability. To decide if global and/or local structures should be interpreted and thus retained in sPCA analyses, we used the global and local tests implemented in *adegenet* as proposed by Jombart (2008). The first three retained axes where then interpolated on 10-m resolution grids covering our study area, and the resulting rasters used to create an RGB composite, using the Merge function in QGIS 3.4 (see example scripts here: https://github.com/rojaff/LanGen_pipeline). The resulting colour patterns represent the similarity in adaptive genetic composition. Finally, to visualize admixture in neutral genetic clusters, we calculated ancestry coefficients using the *snmf* function from the LEA package and plotted them for each individual, as in classical STRUCTURE-like figures (Frichot and François 2015).

Aiming to characterize genetic diversity within each demographic unit, the following metrics were calculated for each neutral genetic cluster: expected heterozygosity (*H*_E_), inbreeding coefficients (*F*), nucleotide diversity (π) and effective population size (*N*_e_). While the former three were calculated using the ‘het’ option in VCFtools implemented in *r2vcftools* (Danecek *et al.* 2011), *N*_e_ was estimated employing the LD approach of NeEstimator 2.0.1, with the lowest allele frequency value set to 0.05 (Do *et al.* 2014). Pairwise genetic differentiation between genetic clusters (*F*_ST_) was also estimated using *r2vcftools*. Additionally, we calculated Tajima’s *D*, representing the difference between the mean number of pairwise differences and the number of segregating sites. Negative values of Tajima’s *D* show that a cluster has an excess of rare alleles, indicating a recent selective sweep or a population expansion after a recent bottleneck. Positive values appear when rare alleles are lacking, therefore suggesting balancing selection or a sudden population contraction. In a population of constant size evolving under mutation-drift equilibrium Tajima’s *D* is expected to be zero (Tajima 1989). Genome-wide estimates of Tajima’s *D* were computed using *r2vcftools* package, which performs a simulation from the neutral model to correct for bias due to the minor-allele-frequency filter.

### Identification of putative adaptive loci and adaptive genetic structure

Several environmental variables have been identified as drivers of local adaptation in plants, including climatic factors like temperature, precipitation and radiation (Eckert *et al.* 2010; Manel *et al.* 2012; De Kort *et al.* 2014), as well as soil characteristics (Supple *et al.* 2018). Lacking high-resolution soil layers for our study region, here we could only assess genotype associations with the commonly employed bioclimatic variables (http://www.worldclim.org/bioclim). We selected a set of orthogonal environmental variables, running a PCA on all 19 WorldClim bioclimatic variables plus elevation (Fick and Hijmans 2017). The variables showing the strongest correlation with the first three PCA axes (85 % of total variance explained) were selected (Minimum Temperature of the Coldest Month, Maximum Temperature of the Warmest Month and Precipitation of the Wettest Quarter) to perform environmental association tests. We then ran latent factor mixed models (LFMM), employing the original genomic data set filtered only by quality and depth, but not for LD (as LFMM remove the effect of relatedness and genetic linkage when inferring ecological associations; François *et al.* 2016) nor for HWE (as loci under selection are expected to depart from HWE). Latent factor mixed models have been used extensively and are currently one of the most commonly used Environmental Association Analysis approaches (Ahrens *et al.* 2018), since they provide a good compromise between detection power and error rates, and are robust to a variety of sampling designs and underlying demographic models (Rellstab *et al.* 2015). Latent factor mixed models were implemented using the *lfmm* function from the *LEA* package (Frichot and François 2015) with *k* = 3 latent factors, where *k* was the optimal number of ancestral populations detected (see results). We used 1000 iterations, a burn-in of 10 000 and 10 runs per environmental variable (Frichot and François 2015). The *P*-values were adjusted using the genomic inflation factor (λ) and false discovery rates were set using the Benjamini–Hochberg algorithm at a rate of *q* = 0.05 (François *et al.* 2016). We then ran the same methods described above to assess neutral genetic structure (DAPC and sPCA) but this time employed only putative adaptive SNPs identified with LFMM. In order to visualize genotype–environment associations, we performed a redundancy analysis (RDA) using only the candidate loci identified in LFMM and the same three climatic variables. To do so we first imputed missing genotypes (20 %) based on population assignments, using *snmf* and the *impute* function from the *LEA* package. Redundancy analysis was then performed using the *rda* function from the Vegan package (Oksanen *et al.* 2019), and the first two constrained axes were plotted using different colours to represent Canga plateaus.

We further investigated the functions of putative adaptive loci. Sequences (RAD contigs) containing putative adaptive SNPs were submitted to the EMBOSS Transeq (http://www.ebi.ac.uk/Tools/st/emboss_transeq/) to obtain the corresponding protein sequences coded by genes contained in the flanking regions of our candidate SNPs. We used all six frames with standard code (codon table), regions (start–end), trimming (yes) and reverse (no). We then ran a functional analysis using InterPro (https://www.ebi.ac.uk/interpro/; Mitchell *et al.* 2018), searching for GO terms and pathways along the respective annotation databases (Interpro, Pfam, Tigrfam, Prints, PrositePattern and Gene3d).

## Results

**Figure 3.**
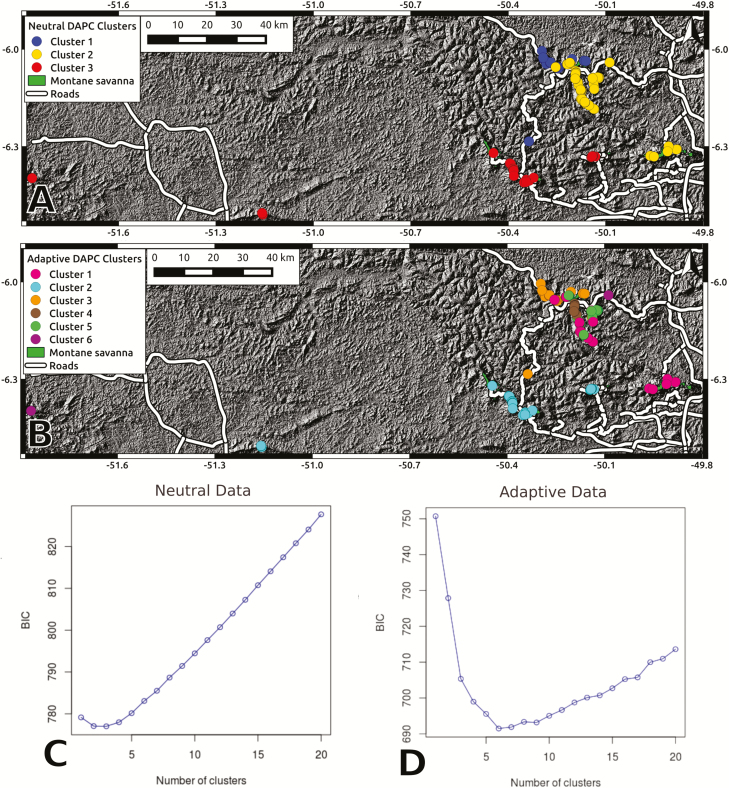
Discriminant analysis of principal component genetic cluster assignments for neutral (A) and putative adaptive (B) markers against a hillshade elevation map (from USGS Earth Explorer) and roads (from IBGE and Vale SA). Bayesian Information Criterion plots show the optimal number of neutral (C) and adaptive (D) genetic clusters.

**Figure 4.**
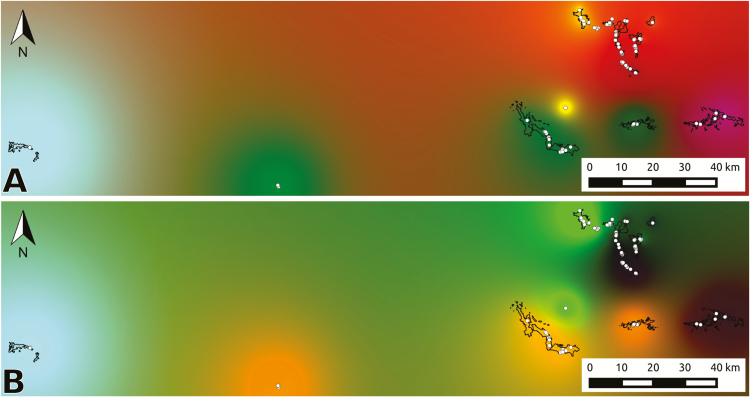
Spatial patterns of neutral (A) and adaptive (B) genetic variation. Maps show the resulting RGB composites created from the interpolated spatial principal component analysis components, and samples are shown as white dots. Areas with similar colours represent similar neutral or adaptive genetic composition.

From a total of 9365 SNPs identified in the 150 sampled individuals, we kept 1911 neutral SNPs after filtering for quality, depth, LD, HWE and *F*_ST_ outlier loci. Neutral genetic structure assessed through DAPC revealed three genetic clusters (Fig. 3A), whereas a more pronounced spatial structure was observed when interpolating the first three spatial principal components (sPCA; Fig. 4A). While effective population sizes were below 100 in all genetic clusters (ranging from 49 to 77), none revealed significant inbreeding (Table 1). Cluster 2 showed negative Tajima’s *D* values, indicative of a population expansion following a recent bottleneck (Table 1). Pairwise genetic differentiation (*F*_ST_) between neutral clusters ranged between 0.024 and 0.048 and substantial admixture was detected **[see****Supporting Information—Fig. S1****]**.

**Table 1.** Genetic diversity measures for the identified neutral genetic clusters. The number of sampled individuals (*N*) is followed by mean expected heterozygosity (*H*_E_), mean inbreeding coefficient (*F*), mean per-site nucleotide diversity (π), effective population size (*N*_e_) and Tajima’s *D*. All estimates are shown along 95 % confidence intervals (CI).

Clusters	*N*	*H*_E_ (CI)	*F* (CI)	π (CI)	*N*_e_ (CI)	Tajima’s *D* (CI)
Cluster1	24	0.25 (0.25/0.25)	0.00 (−0.08/0.08)	0.16 (0.16/0.17)	72.1 (67.8/76.9)	−0.03 (−0.19/0.14)
Cluster2	89	0.21 (0.21/0.22)	−0.02 (−0.07/0.01)	0.20 (0.19/0.21)	76.9 (75.8/78.0)	−0.34 (−0.52/−0.16)
Cluster3	37	0.24 (0.24/0.24)	0.06 (−0.01/0.13)	0.19 (0.19/0.20)	48.7 (47.3/50.1)	0.07 (−0.04/0.26)

**Figure 5.**
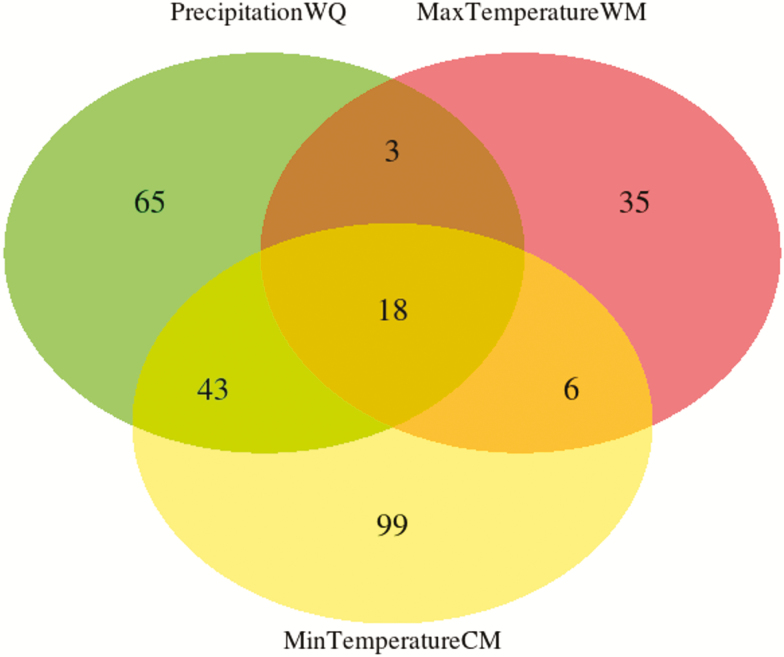
Venn diagram showing the number of sequences (RAD contigs) containing candidate SNPs associated with each environmental variable (Minimum Temperature of Coldest Month in yellow, Maximum Temperature of Warmest Month in red and Precipitation of Wettest Quarter in green) or with more than one variable (values in intersections).

Nearly 10 % of all analysed sequences contained putative adaptive SNPs (Table 2), most of them associated with minimum temperature of the coldest month, while 18 sequences contained SNPs associated with all environmental variables (Fig. 5). Adjusted *P*-values for genotype–environment associations ranged between 0.05 and 1 × 10^−8^, and revealed stronger associations with precipitation of the wettest quarter and minimum temperature of the coldest month **[see****Supporting Information—Fig. S2****]**. Only 25 sequences contained InterPro annotations and two annotated genes were shared between all environmental variables (Table 3). Adaptive genetic structure assessed through DAPC revealed six genetic clusters (Fig. 3B), and a less pronounced spatial structure was found when using the sPCA approach (Fig. 4B). Redundancy analysis revealed that 18.8 % of adaptive variation was associated with climatic variables (*P* = 0.001), with individuals from Serra Norte and Bocaina showing genotype associations with higher temperatures and higher precipitation levels **[see****Supporting Information—Fig. S3****].**

**Table 2.** Number of adaptive signals detected employing environmental association tests. The number of candidate SNPs and sequences (RAD contigs) containing candidate SNPs are shown for each environmental variable. Numbers in parentheses represent independent (non-overlapping) detections. ^a^Genomic inflation factor (λ) = 1.45; ^b^λ = 0.65; ^c^λ = 1.5.

Signal type	Total analysed	Total under selection	Environmental association tests		
			Precipitation WQ^a^	Max temperature WM^b^	Min temperature CM^c^
SNPs	9253	768	385 (181)	174 (82)	501 (273)
RAD contigs	2547	269	129 (65)	62 (35)	166 (99)

**Table 3.** Annotated putative adaptive proteins. *Proteins also identified as putative adaptive in other plant species from Carajás (Lanes *et al.* 2018).

Climatic variable	Signature description
Precipitation of Wettest Quarter	Serine-threonine/tyrosine-protein kinase catalytic domain*
	Papain family cysteine protease, peptidase C1A, papain C-terminal
	No apical meristem (NAM) protein, NAC domain
	Homeobox-associated leucine zipper
	NAD(P)-binding domain*
	Phosphoesterase
	Fatty acid desaturase domain
Max Temperature of Warmest Month	Chloramphenicol acetyltransferase-like domain*
	Transferase
	FAD-linked oxidase, N-terminal, FAD-binding domain*
	CO dehydrogenase flavoprotein-like, FAD-binding, subdomain 2
	Squalene epoxidase
	FAD/NAD(P)-binding domain*
	Homeobox-associated leucine zipper
	Serine-threonine/tyrosine-protein kinase catalytic domain*
Min Temperature of Coldest Month	Homeobox-associated leucine zipper
	Enolase C-terminal domain-like
	Alpha/beta hydrolase fold
	Chloramphenicol acetyltransferase-like domain*
	Transferase
	Photosystem II Psb28, class 1
	Glutathione S-transferase, C-terminal-like
	Serine-threonine/tyrosine-protein kinase catalytic domain*
	No apical meristem (NAM) protein, NAC domain
	Sodium:solute symporter family

## Discussion

This study assessed genetic diversity, neutral and adaptive genetic structure in 150 individuals from *B. carajensis* collected across the species’ currently known distribution range. While neutral genetic structure indicated the presence of at least three discrete genetic clusters, six adaptive genetic clusters were found. Despite the small estimated effective population sizes, genetic diversity was similar to that found in other plant species occurring in the region, and none of the neutral clusters showed significant inbreeding. In addition, around 10 % of the analysed sequences contained putative adaptive SNPs associated with environmental variables, revealing different selective pressures in a narrowly distributed species. Below we discuss our findings, and show how combining information derived from neutral and adaptive genomic markers can help guide conservation efforts.

Levels of genetic diversity observed for *B. carajensis* were generally higher or similar to those observed for other patchily distributed herbs, shrubs and trees (Mosca *et al.* 2012; Jennings *et al.* 2016; Lanes *et al.* 2018; Carvalho *et al.* 2019; Resende-Moreira *et al.* 2019). Heterozygosity and nucleotide diversity were similar or higher than those found in two sympatric morning glories (Lanes *et al.* 2018), although effective population sizes were an order of magnitude lower in *B. carajensis*. Despite these small effective population sizes (all below 100), the lack of inbreeding, the low levels of genetic differentiation and the existence of admixture among the studied populations suggest there is no short-term risk of inbreeding depression (Kirk and Freeland 2011; Jamieson and Allendorf 2012).

Our study reveals at least three neutral genetic clusters, generally associated with multiple Montane Savanna plateaus (Figs 3A and 4A). In contrast, neutral genetic structure was strongly associated with Canga plateaus in a sympatric but broadly distributed morning glory (Lanes *et al.* 2018). Since Tajima’s *D* values did not depart from zero in clusters 1 and 3, suggesting constant population sizes evolving under mutation-drift equilibrium, we cannot attribute the spatial pattern of cluster 2 to an ancient population expansion (as suggested by the negative Tajima’s *D* values) (Sabeti *et al.* 2006). The fact that all neutral genetic clusters span multiple Canga plateaus, together with the high observed admixture among genetic clusters **[see****Supporting Information—Fig. S1****]** and low pairwise *F*_ST_ values, suggest long-distance dispersal. Indeed, a recent landscape genomic study found that gene flow in *B. carajensis* is mainly influenced by geographic distance, implying that forested areas surrounding the plateaus do not represent important barriers to gene flow (Carvalho *et al.* 2019).

Wind dispersal is one of the most common long-distance dispersal mechanisms, often driven by extreme events such as rare weather conditions (Soons *et al.* 2004). Montane Savanna highlands from Carajás are characterized by strong winds, which could certainly facilitate the colonization of distant plateaus by *B. carajensis* as well as enhance genetic connectivity between them (Nathan 2006; Carvalho *et al.* 2019). For instance, a study investigating another bee-pollinated Melastome species found long-distance pollen dispersal, reaching up to 3 km (Castilla *et al.* 2017). In addition, anthropogenic interference could also contribute to long-distance dispersal events in our studied species. There are many examples of humans acting as both active and passive plant dispersal vectors, allowing different types of seeds to reach very long distances and resulting in introgression (Auffret *et al.* 2014; Bullock *et al.* 2018). The small and abundant seeds of the *B. carajensis* (Almeda *et al.* 2016) are resistant to desiccation (they survive throughout the dry season), and can germinate in different substrates, characteristics that increase the likelihood of human-mediated dispersal (Bullock *et al.* 2018; Carvalho *et al.* 2019). Moreover, Canga plateaus are connected by roads and there is a frequent transit of vehicles in the area (Fig. 3A), which could facilitate human-mediated gene flow through the transportation of seeds in shoes or car tires (Von Der Lippe and Kowarik 2007; Wichmann *et al.* 2009).

Nearly 10 % of our analysed sequences contained candidate loci showing environmental associations (Table 2), a similar proportion to that found in previous studies (Ahrens *et al.* 2018). Using these putative adaptive loci we found six genetic clusters (AU), revealing different selective pressures across the specie’s distribution range (Figs 3B and 4B; **see****Supporting Information—Fig. S3**). Such a stark adaptive genetic structure was unexpected given the narrow environmental gradients found within Canga ecosystems **[see****Supporting Information—Fig. S4****]**, the small effective population sizes and the high gene flow between populations (Funk *et al.* 2012). Our results thus show that the climatic variability captured by bioclimatic layers was enough to detect non-random genotype–environment associations, but suggest that many more associations are likely to be detected when high-resolution climatic and soil data are made available. The small effective population sizes, on the other hand, may reflect the specie’s life history characteristics, and thus be sufficient to assure enough genetic variation for local adaptation to occur. Finally, local adaptation under high gene flow has been shown in other organisms (Godbout *et al.* 2019; Krohn *et al.* 2019), and suggest strong selective pressures associated with local environments are constantly shaping standing genetic variation (Allendorf *et al.* 2013).

Our environmental association tests can be considered conservative, because they accounted for false discovery rates (François *et al.* 2016) as well as the underlying demographic structure (Frichot and François 2015). We nevertheless note that other genes occurring in the flanking regions of our candidate SNPs could be responsible for the detected adaptive signals, and that many sequences did not match translated proteins, or found matches with uncharacterized proteins. Despite these methodological limitations, we found candidate genes coding proteins that play a variety of important roles related to plant metabolism, growth, development, stress tolerance, cellular transport and regulation of physiological functions **[see****Supporting Information—Table S1****]**. Two proteins seem to be of especial importance, given they were associated with all environmental variables (Table 3): the serine-threonine/tyrosine-protein kinase catalytic domain regulates cellular and metabolic events (Parthibane *et al.* 2012), while the homeobox-associated leucine zipper is an important DNA-binding transcription factor regulating development and morphology in different plant organs (Chan *et al.* 1998; Jain *et al.* 2008). These proteins act at early and late developmental stages and have been associated with responses to different types of stresses, including water deficit, pathogens, salt concentration and cold temperatures (Chan *et al.* 1998; Zhang and Klessig 2001; Jain *et al.* 2008), as well as with other climatic responses (Ahrens *et al.* 2019; Ferrero-Serrano and Assmann 2019). In addition, five proteins (Table 3) were also found as putative adaptive in two morning glories from Carajás (Lanes *et al.* 2018), some of which are also involved in stress responses. We nevertheless note that functional validation of these candidate proteins is still necessary to confirm their role in local adaptations.

### Conservation implications

Our results reveal that *B. carajensis* is structured in at least three neutral genetic clusters and six adaptive genetic clusters, which could be considered MU and AU, respectively. Since the recently created Campos Ferruginosos National Park (Fig. 2) is already protecting a portion of each of our identified MU (Fig. 3A), there seems to be no imminent risk of losing any of these MU. Admixture between MU and the lack of inbreeding suggest there is no short-term risk of inbreeding depression, although possible gene flow reductions caused by further habitat loss and fragmentation would make these units susceptible to genetic drift, considering their small effective population sizes (Jamieson and Allendorf 2012). On the other hand, not all AU are currently protected, and some are located in the vicinity of mining operations. Management options to prevent losing the adaptive genetic variation found in these AU include *in situ* or *ex situ* actions. In the later case, a sample of individuals from an AU could be either re-located or preserved through seed banks. Following the recommendations made by Frankham *et al.* (2017, page 216), genetic clusters should be managed separately when they show both neutral and adaptive differentiation (in our case AU from different MU), as risks of outbreeding depression are usually high. Under adaptive differentiation with no neutral differentiation (in our case AU within each MU), genetic rescue is only recommended when the risk of outbreeding depression is low. We believe this is the case in our study system, given that environmental differences are small and gene flow is high between (Fig. 3) and within each MU (Carvalho *et al.* 2019). Our results thus suggest that neutral genetic clusters (MU) should be managed separately, and that possible genetic rescue or re-location efforts should only employ individuals sampled within each of our identified MU.

Our study exemplifies how assumption-free genetic clustering methods and environmental association tests applied to a large genomic data set can be employed to delineate MU and AU, and thereby inform management decisions to prevent the loss of unique genetic variation and maximize species resilience to future environmental change. This approach is particularly useful to inform management decisions of rare and endangered species for which it is difficult or impossible to assess local adaptations using traditional common garden or reciprocal transplant experiments.

## Supporting Information

The following additional information is available in the online version of this article—

**Table S1.** Functions of candidate proteins encoded by genes contained in the flanking regions of candidate SNPs.

**Figure S1.** Plots displaying ancestry coefficients retrieved from sparse non-negative matrix factorization (*snmf*) using neutral markers.

**Figure S2.** Manhattan plots of log-transformed adjusted *P*-values of candidate SNPs for each environmental variable, generated by latent factor mixed models (LFMM).

**Figure S3.** Triplot showing axes 1 and 2 of redundancy analysis (RDA) using the putative adaptive SNPs identified with latent factor mixed models (LFMM).

**Figure S4.** Variation in the three climatic variables used in environmental association tests (latent factor mixed models, LFMM) across the studied region.

plaa003_suppl_Supplementary_Figures

plaa003_suppl_Supplementary_Tables

## Data

Genotypes, sequences and geographic coordinates for all samples are publicly available in FigShare: https://doi.org/10.6084/m9.figshare.8224175.v3
