# Supplementary material for: Range-wide neutral and adaptive genetic structure of an endemic herb from Amazonian Savannas
Source: AoB Plants. 2020 Jan 31;12(1):plaa003. doi: 10.1093/aobpla/plaa003 (PMC7043808; doi:10.1093/aobpla/plaa003)
Supplement: plaa003_suppl_Supplementary_Figures [file plaa003_suppl_supplementary_figures.pdf]

Supporting Information

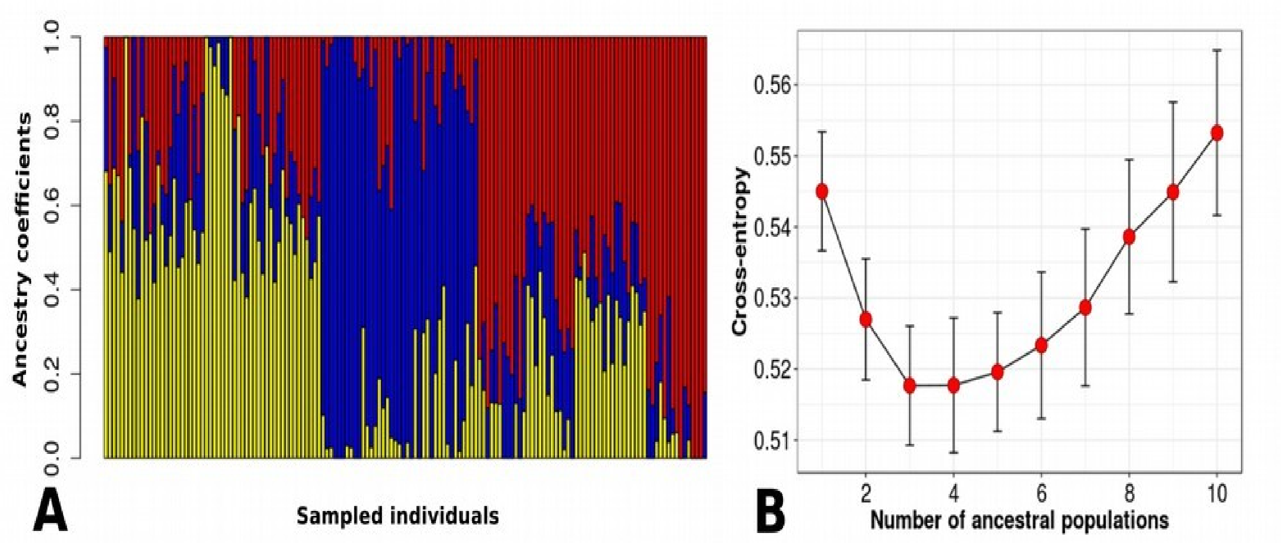

Figure S1: A: Ancestry coefficients retrieved from sparse non-negative matrix factorization (*snmf*) using neutral markers. Colors represent the different genetic clusters (and match the colors used in Fig. 3A). B: Plot showing the optimal number of ancestral populations (k) based on mean ± sd cross-entropy.

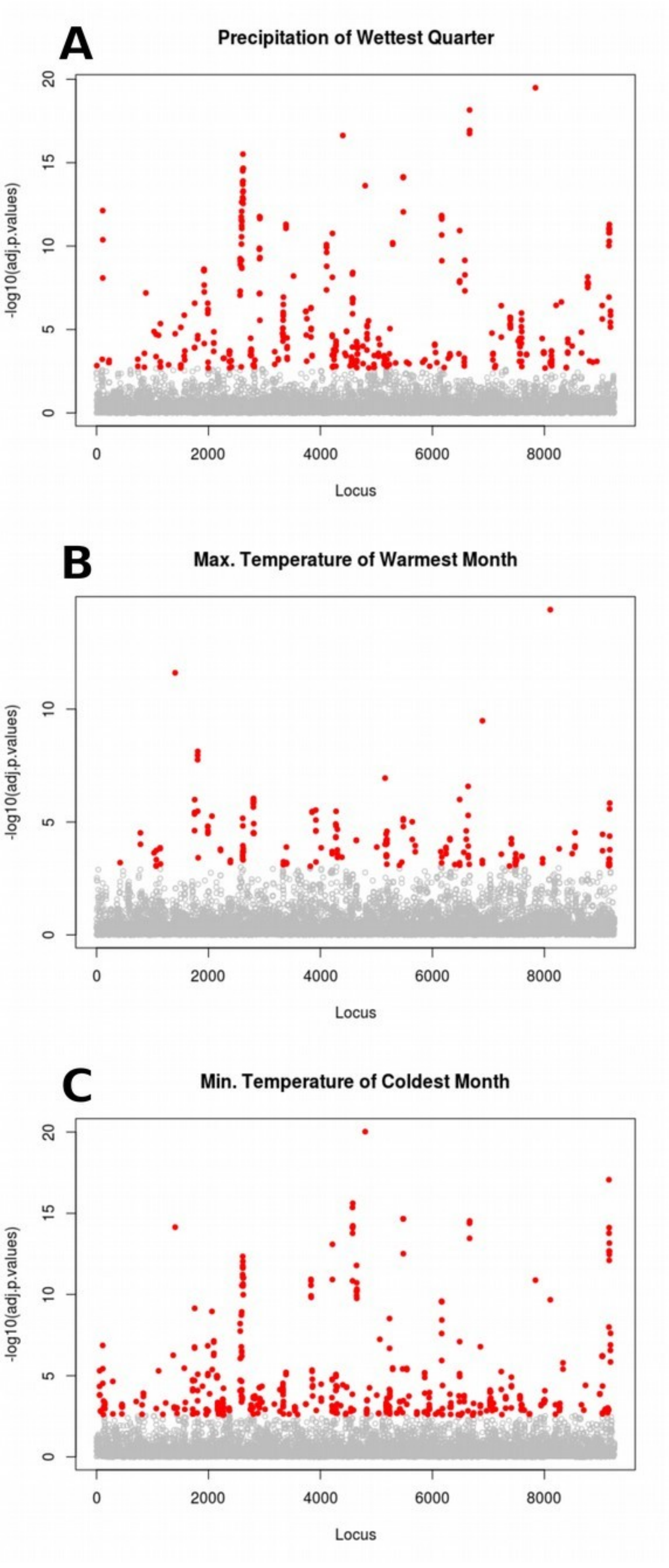

Figure S2: Manhattan plots of log-transformed adjusted *P*-values of candidate SNPs for each environmental variable, generated by latent factor mixed models (LFMM). Candidate SNPs selected with adjusted P-values < 0.05 are shown in red.

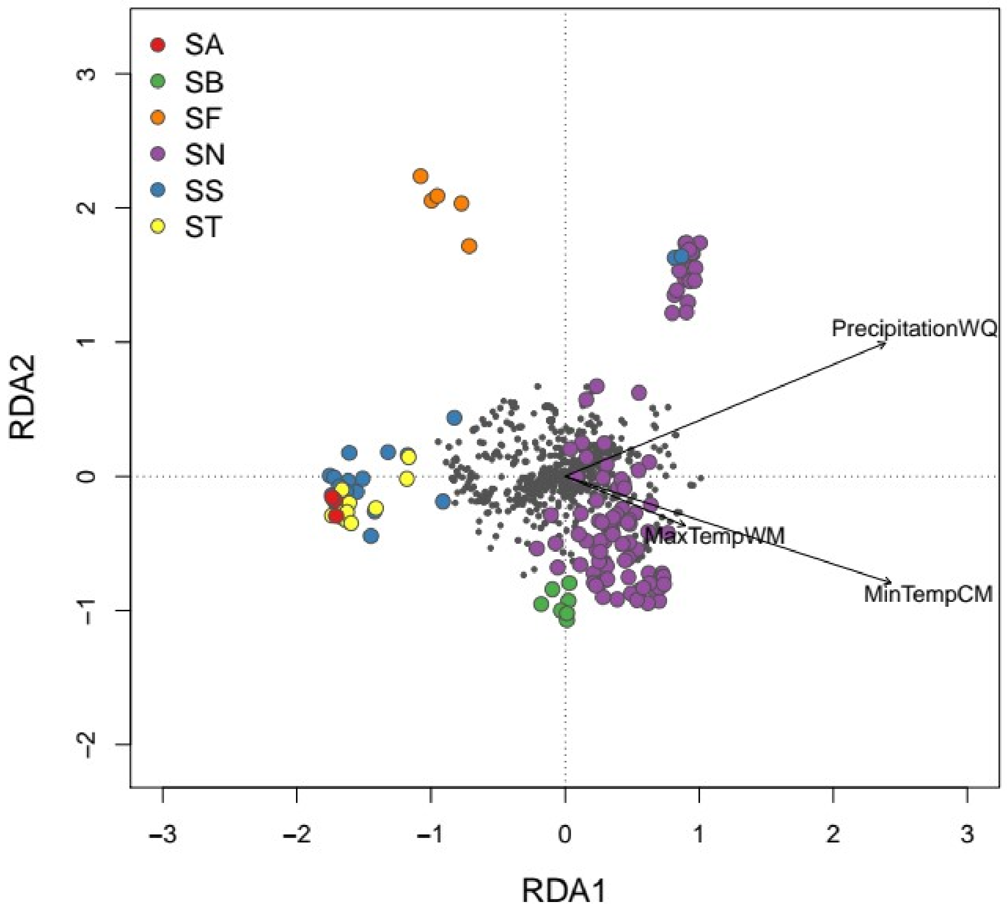

Figure S3: Triplot showing axes 1 and 2 of redundancy analysis (RDA) using the putative adaptive SNPs identified with LFMM. Grey points are SNPs, colors indicate sampling locations and vectors represent environmental predictors. PrecipitationWQ - Precipitation of Wettest Quarter; MinTempCM - Min Temperature of Coldest Month; MaxTempWM - Max Temperature of Warmest Month; SA- Serra Arqueada ; SB- Serra da Bocaina; SF - São Félix; SN - Serra Norte; SS - Serra Sul; ST - Serra do Tarzan.

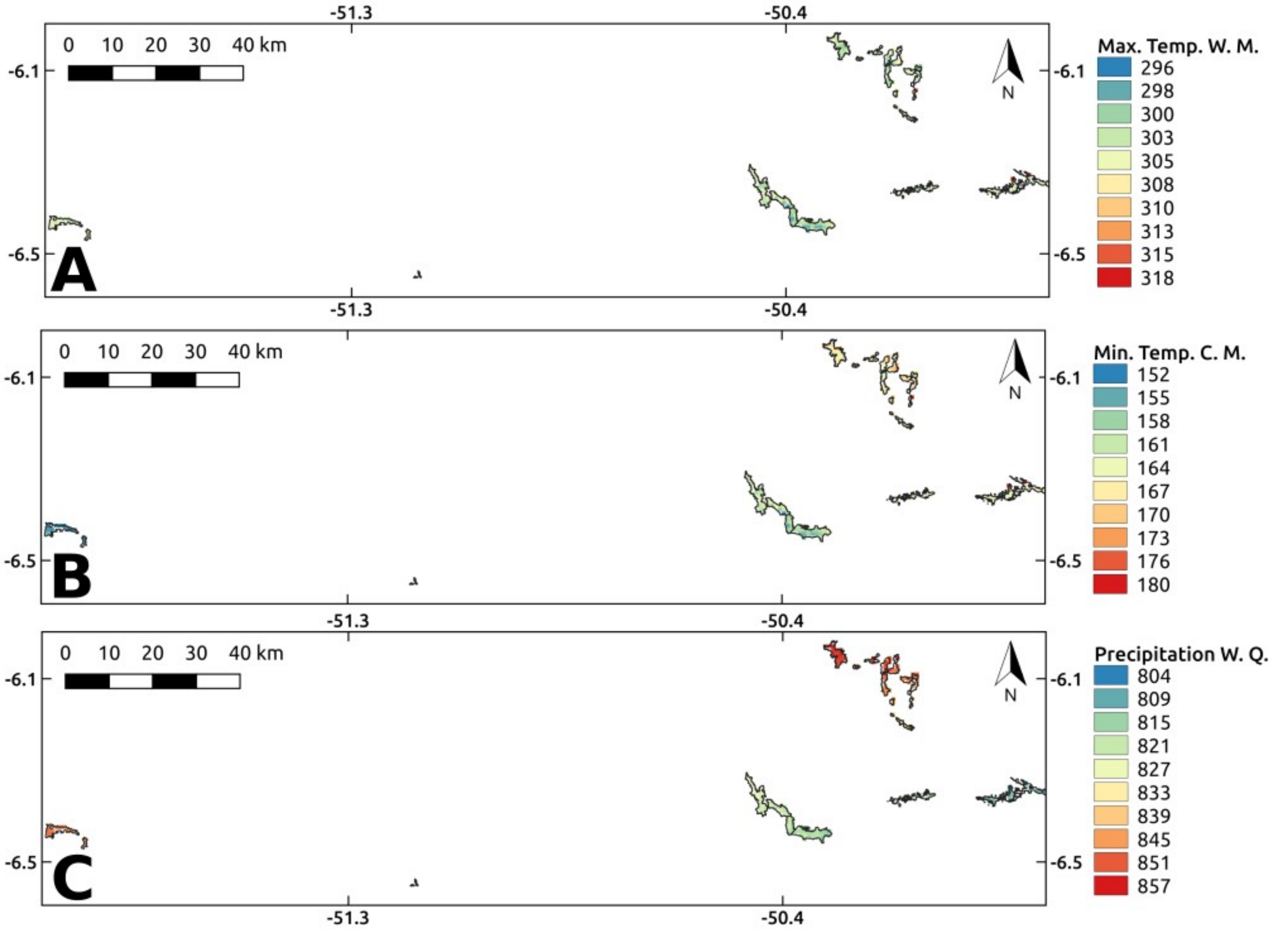

Figure S4: Maps showing variation in the three climatic variables used to run environmental association tests (LFMM) across the main Canga plateaus from our study region. (A) Maximum Temperature of Warmest Month; (B) Minimum Temperature of Coldest Month and (C) Precipitation of Wettest Quarter.
