# Supplementary material for: Range-wide neutral and adaptive genetic structure of an endemic herb from Amazonian Savannas
Source: AoB Plants. 2020 Jan 31;12(1):plaa003. doi: 10.1093/aobpla/plaa003 (PMC7043808; doi:10.1093/aobpla/plaa003)
Supplement: plaa003_suppl_Supplementary_Tables [file plaa003_suppl_supplementary_tables.pdf]

Supporting Information

Table S1: Functions of candidate proteins encoded by genes contained in the flanking regions of candidate SNPs.

Climatic variable	Signature description	InterPro accession	Possible functions in plants	References
Precipitation of Wettest Quarter	Serine-threonine/ tyrosine-protein kinase catalytic domain	IPR001245	Regulation of cellular and metabolic events	(Zhang and Klessig 2001; Rudrabhatla et al. 2006; Opdenakker et al. 2012; Parthibane et al. 2012)
	Papain family cysteine protease, Peptidase C1A, papain C-terminal	IPR000668	Regulation of physiological functions (e.g. senescence and seed germination)	(Martínez et al. 2012)
	No apical meristem (NAM) protein, NAC domain	IPR003441	Involved in developmental processes, including formation of apical meristem, lateral shoots and floral organs, as well as hormonal control and response to biotic and abiotic stress	(Souer et al. 1996; Aida et al. 1997; Kikuchi et al. 2000; Xie et al. 2000; Duval et al. 2002; Hegedus et al. 2003; Olsen et al. 2005)
	Homeobox associated leucine zipper	IPR003106	DNA binding transcription factor	(Schena and Davis 1994; Chan et al. 1998; Jain et al. 2008; Vlad et al. 2014)
	NAD(P)-binding domain	IPR016040	Present in different proteins associated to NAD(P) regulated functions, important in redox homeostasis, transcriptional regulation, microtube metabolism	(Hashida et al. 2009)
	Phosphoesterase	IPR007312	Cleaves phosphoester bonds. Variable functions related to nucleic acid metabolism and cellular signaling, including DNA repair, recycling of cAMP and RNA processing and gene expression control	(Assmann 1995; Betti et al. 2001; Mazumder et al. 2002)
	Fatty acid desaturase domain	IPR005804	Unsaturated fatty acid production, cellular membrane function, tolerance to various environmental stresses, especially temperature stresses	(Los and Murata 1998; Iba 2002)
Max Temperature of Warmest Month	Chloramphenicol acetyltransferase-like domain	IPR023213	Tranferase activity, similar to BADH acyltransferase, a large family of enzimes that use acyl CoA and produce small volatile esters, modified anthocyanins, defense	(D'Auria 2006)

			compounds and phytoalexins	
	Transferase	IPR003480	Transfer a specific functional group from one molecule to another. Variable functions including light signaling, development modulation, protection against oxidative damage, tolerance to abiotic and biotic stress, etc.	(Dixon et al. 2002; Hartweck et al. 2002; Sappl et al. 2009; Chen et al. 2012; Domitrovic et al. 2017; Gullner et al. 2018)
	FAD linked oxidase, N-terminal, FAD binding domain	IPR006094	FAD-meadiated oxidation of a variety of substrates, Energy metabolism, apoptosis, maintenance of redox homoeostasis, and cellular signaling	(Dong et al. 2016)
	CO dehydrogenase flavoprotein-like, FAD-binding, subdomain 2	IPR016169	CO oxidation	(Meyer et al. 2000; Fukuyama 2004)
	Squalene epoxidase	IPR013698	Production of plant secondary metabolites (triterpenoids).Converts squalene into the precursor of angiosmerm triterpenoids, a secondary metabollite associated with a variety of fuctions(membrane sterols, phytormones, saponins, defense compounds, etc)	(Phillips et al. 2006; Rasbery et al. 2007)
	FAD/NAD(P)-binding domain	IPR023753	Oxidation-reduction process	(Hyde et al. 1991; Eppink et al. 1997; Ojha et al. 2007)
	Homeobox associated leucine zipper	IPR003106	DNA binding transcription factor	(Schena and Davis 1994; Chan et al. 1998; Jain et al. 2008; Vlad et al. 2014)
	Serine-threonine/ tyrosine-protein kinase catalytic domain	IPR001245	Regulation of cellular and metabolic events	(Zhang and Klessig 2001; Rudrabhatla et al. 2006; Opdenakker et al. 2012; Parthibane et al. 2012)
Min Temperature of Coldest Month	Homeobox associated leucine zipper	IPR003106	DNA binding transcription factor	(Schena and Davis 1994; Chan et al. 1998; Jain et al. 2008; Vlad et al. 2014)
	Enolase C-terminal domain-like	IPR029065	Glycolytic process, conversion of 2-phosphoglycerate to phosphoenolpyruvate in the glycolytic pathway	(Van Der Straeten et al. 1991; Gerlt et al. 2005)
	Alpha/Beta hydrolase fold	IPR029058	Variable enzymatic catalysis	(Carr and Ollis 2009)
	Chloramphenicol acetyltransferase-like domain	IPR023213	Tranferase activity, similar to BADH acyltransferase, a large family of enzimes that use acyl CoA	(D’Auria 2006)

			and produce small volatile esters, modified anthocyanins, defense compounds and phytoalexins	
	Transferase	IPR003480	Transfer a specific functional group from one molecule to another. Variable functions including light signaling, development modulation, protection against oxidative damage, tolerance to abiotic and biotic stress, etc.	(Dixon et al. 2002; Hartweck et al. 2002; Sappl et al. 2009; Chen et al. 2012; Domitrovic et al. 2017; Gullner et al. 2018)
	Photosystem II Psb28, class 1	IPR005610	Photosyntesis; repair of photoinhibitory damage	(Hagman et al. 1997; Shi and Schröder 2004)
	Glutathione S-transferase, C-terminal-like	IPR010987	Cellular transport, interacts with others compounds to activate systemic acquired resistance to pathogens	(Banday and Nandi 2018)
	Serine-threonine/ tyrosine-protein kinase catalytic domain	IPR001245	Regulation of cellular and metabolic events	(Zhang and Klessig 2001; Rudrabhatla et al. 2006; Opdenakker et al. 2012; Parthibane et al. 2012)
	No apical meristem (NAM) protein, NAC domain	IPR003441	Involved in developmental processes, including formation of apical meristem, lateral shoots and floral organs, as well as hormonal control and response to biotic and abiotic stress	(Souer et al. 1996; Aida et al. 1997; Kikuchi et al. 2000; Xie et al. 2000; Duval et al. 2002; Hegedus et al. 2003; Olsen et al. 2005)
	Sodium:solute symporter family	IPR001734	Solute transport	(Jung 2002; Wang et al. 2008)

References

Aida M, Ishida T, Fukaki H, et al (1997) Genes lnvolved in Organ Separation in Arabidopsis: An Analysis of the cup-shaped cotyledon Mutant. Plant Cell 9:841–857

Assmann SM (1995) Cyclic AMP as a Second Messenger in Higher Plants (status and future prospects). Plant Physiol 108:885–889

Banday ZZ, Nandi AK (2018) Arabidopsis thaliana GLUTATHIONE- S -TRANSFERASE THETA 2 interacts with RSI1/FLD to activate systemic acquired resistance. Mol Plant Pathol 19:464–475. doi: 10.1111/mpp.12538

Betti M, Petrucco S, Bolchi A, et al (2001) A Plant 3’-Phosphoesterase Involved in the Repair of DNA Strand Breaks Generated by Oxidative Damage. J Biol Chem 276:18038–18045. doi: 10.1074/jbc.M010648200

Carr PD, Ollis DL (2009) α/β Hydrolase Fold: An Update. Protein Pept Lett 16:1137–1148

Chan RL, Gago GM, Palena CM, Gonzalez DH (1998) Homeoboxes in plant development. Biochim Biophys Acta - Gene Struct Expr 1442:1–19. doi: 10.1016/S0167-4781(98)00119-5

Chen J, Jiang H, Hsieh E, et al (2012) Drought and Salt Stress Tolerance of an Arabidopsis Glutathione S -Transferase U17 Knockout Mutant Are Attributed to the Combined Effect of Glutathione and Abscisic Acid. Plant Physiol 158:340–351. doi: 10.1104/pp.111.181875

D’Auria JC (2006) Acyltransferases in plants: a good time to be BAHD. Curr Opin Plant Biol 9:331–340. doi: 10.1016/j.pbi.2006.03.016

Dixon DP, Lapthorn A, Edwards R (2002) Plant glutathione transferases. Genome Biol 3:1–10

Domitrovic T, Fausto AK, Silva T da F, et al (2017) Plant arginyltransferases (ATEs). Genet Mol Biol 40:253–260

Dong C, Cao N, Zhang Z, Shang Q (2016) Characterization of the Fatty Acid Desaturase Genes in Cucumber: Structure, Phylogeny, and Expression Patterns. PLoS One 11:1–22. doi: 10.1371/journal.pone.0149917

Duval M, Hsieh T, Kim SY, Thomas TL (2002) Molecular characterization of AtNAM: a member of the Arabidopsis NAC domain superfamily. Plant Mol Biol 50:237–248

Eppink MHM, Schreuder HA, Berkel WJH Van (1997) Identification of a novel conserved sequence motif in flavoprotein hydroxylases with a putative dual function in FAD/NAD (P) H binding. Protein Sci 6:2454–2458

Fukuyama K (2004) Structure and function of plant-type ferredoxins. Photosynth Res 81:289–301

Gerlt JA, Babbitt PC, Rayment I (2005) Divergent evolution in the enolase superfamily: the interplay of mechanism and specificity. Arch Biochem Biophys 433:59–70. doi: 10.1016/j.abb.2004.07.034

Gullner G, Komives T, Király L, Schröder P (2018) Glutathione S-Transferase Enzymes in Plant-Pathogen Interactions. Front Plant Sci 9:1–19. doi: 10.3389/fpls.2018.01836

Hagman A, Shi L, Rintamäki E, et al (1997) The Nuclear-Encoded PsbW Protein Subunit of Photosystem II Undergoes Light-Induced Proteolysis. Biochemistry 36:12666–12671

Hartweck LM, Scott CL, Olszewski NE (2002) Two O-Linked N-Acetylglucosamine Transferase Genes of Arabidopsis thaliana L. Heynh. Have Overlapping Functions Necessary for Gamete and Seed Development. Genetics 161:1279–1291

Hashida S, Takahashi H, Uchimiya H (2009) The role of NAD biosynthesis in plant development and stress responses. Ann Bot 103:819–824. doi: 10.1093/aob/mcp019

Hegedus D, Yu M, Baldwin D, et al (2003) Molecular characterization of Brassica napus NAC domain transcriptional activators induced in response to biotic and abiotic stress. Plant Mol Biol 53:383–397

Hyde GE, Crawford NM, Campbell. WH (1991) The Sequence of Squash NADH:Nitrate Reductase and Its Relationship to the Sequences of Other Flavoprotein Oxidoreductases. J Biol Chem 266:23542–23547

Iba K (2002) Acclimative response to temperature stress in higher plants: approaches of gene engineering for temperature tolerance. Annu Rev Plant Biol 53:225–245. doi: 10.1146/annurev.arplant.53.100201.160729

Jain M, Tyagi AK, Khurana JP (2008) Genome-wide identification, classification, evolutionary expansion and expression analyses of homeobox genes in rice. FEBS J 275:2845–2861. doi: 10.1111/j.1742-4658.2008.06424.x

Jung H (2002) The sodium/substrate symporter family: structural and functional features. FEBS Lett 529:73–77

Kikuchi K, Ueguchi-Tanaka M, Yoshida KT, et al (2000) Molecular analysis of the NAC gene family in rice. Mol Gen Genet MGG 262:1047–1051

Los DA, Murata N (1998) Structure and expression of fatty acid desaturases. Biochim Biophys Acta 1394:3–15

Martínez M, Cambra I, González Melendi P, et al (2012) C1A cysteine-proteases and their inhibitors in plants. Physiol Plant 145:85–94. doi: 10.1111/j.1399-3054.2012.01569.x

Mazumder R, Iyer LM, Vasudevan S, Aravind L (2002) Detection of novel members, structure-function analysis and evolutionary classification of the 2H phosphoesterase superfamily. Nucleic Acids Res 30:5229–5243

Meyer O, Gremer L, Ferner R, et al (2000) The Role of Se, Mo and Fe in the Structure and Function of Carbon Monoxide Dehydrogenase. Biol Chem 381:865–876

Ojha S, Meng EC, Babbitt PC (2007) Evolution of Function in the "' Two Dinucleotide Binding Domains '" Flavoproteins. PLoS Comput Biol 3:1268–1280. doi: 10.1371/journal.pcbi.0030121

Olsen AN, Ernst HA, Leggio L Lo, Skriver K (2005) NAC transcription factors: structurally distinct, functionally diverse. Trends Plant Sci 10:79–87. doi: 10.1016/j.tplants.2004.12.010

Opdenakker K, Remans T, Vangronsveld J, Cuypers A (2012) Mitogen-Activated Protein (MAP) Kinases in Plant Metal Stress: Regulation and Responses in Comparison to Other Biotic and Abiotic Stresses. Int J Mol Sci 13:7828–7853. doi: 10.3390/ijms13067828

Parthibane V, Iyappan R, Vijayakumar A, et al (2012) Serine/Threonine/Tyrosine Protein Kinase Phosphorylates Oleosin, a Regulator of Lipid Metabolic Functions. Plant Physiol 159:95–104. doi: 10.1104/pp.112.197194

Phillips DR, Rasbery JM, Bartel B, Matsuda SPT (2006) Biosynthetic diversity in plant triterpene cyclization. Curr Opin Plant Biol 9:305–314. doi: 10.1016/j.pbi.2006.03.004

Rasbery JM, Shan H, Leclair RJ, et al (2007) Arabidopsis thaliana Squalene Epoxidase 1 Is Essential for Root and Seed Development. J Biol Chem 282:17002–17013. doi: 10.1074/jbc.M611831200

Rudrabhatla P, Reddy MM, Rajasekharan R (2006) Genome-wide analysis and experimentation of plant serine/ threonine/tyrosine-specific protein kinases. Plant Mol Biol 60:293–319. doi: 10.1007/s11103-005-4109-7

Sappl PG, Carroll AJ, Clifton R, et al (2009) The Arabidopsis glutathione transferase gene family displays complex stress regulation and co-silencing multiple genes results in altered metabolic sensitivity to oxidative stress. Plant J 58:53–68. doi: 10.1111/j.1365-313X.2008.03761.x

Schena M, Davis RW (1994) Structure of homeobox-leucine zipper genes suggests a model for the evolution of gene families. Proc Natl Acad Sci 91:8393–8397

Shi L, Schröder WP (2004) The low molecular mass subunits of the photosynthetic supracomplex, photosystem II. Biochim Biophys Acta 1608:75–96. doi: 10.1016/ j.bbabio.2003.12.004

Souer E, Houwelingen A van, Kloos D, et al (1996) The No Apical Meristem Gene of Petunia Is Required for Pattern Formation in Embryos and Flowers and Is Expressed at Meristem and Primordia Boundaries. Cell 85:159–170

Van Der Straeten D, Rodrigues-Pousada RA, Goodman HM, Van Montagu M (1991) Plant Enolase: Gene Structure, Expression, and Evolution. Plant Cell 3:719–735

Vlad D, Kierzkowski D, Rast MI, et al (2014) Leaf Shape Evolution Through Duplication, Regulatory Diversification, and Loss of a Homeobox Gene. Science (80-) 343:780–784

Wang W, Köhler B, Cao F, Liu L (2008) Molecular and physiological aspects of urea transport in higher plants. Plant Sci 175:467–477. doi: 10.1016/j.plantsci.2008.05.018

Xie Q, Frugis G, Colgan D, Chua N (2000) Arabidopsis NAC1 transduces auxin signal downstream of TIR1 to promote lateral root development. Genes Dev 14:3024–3036. doi: 10.1101/gad.852200.The

Zhang S, Klessig DF (2001) MAPK cascades in plant defense signaling. Trends Plant Sci 6:520–527. doi: 10.1016/S1360-1385(01)02103-3
